# Release of Bacteriocins from Nanofibers Prepared with Combinations of Poly(d,l-lactide) (PDLLA) and Poly(Ethylene Oxide) (PEO)

**DOI:** 10.3390/ijms12042158

**Published:** 2011-03-29

**Authors:** Tiaan Heunis, Osama Bshena, Bert Klumperman, Leon Dicks

**Affiliations:** 1 Department of Microbiology, University of Stellenbosch, 7602 Matieland (Stellenbosch), South Africa; E-Mail: heunistd@sun.ac.za; 2 Department of Chemistry and Polymer Science, University of Stellenbosch, 7602 Matieland (Stellenbosch), South Africa; E-Mails: bshena@sun.ac.za (O.B.); bklump@sun.ac.za (B.K.)

**Keywords:** bacteriocins, electrospinning, nanofiber, antimicrobial activity

## Abstract

Plantaricin 423, produced by *Lactobacillus plantarum*, and bacteriocin ST4SA produced by *Enterococcus mundtii*, were electrospun into nanofibers prepared from different combinations of poly(d,l-lactide) (PDLLA) and poly(ethylene oxide) (PEO) dissolved in *N*,*N*-dimethylformamide (DMF). Both peptides were released from the nanofibers with a high initial burst and retained 88% of their original antimicrobial activity at 37 °C. Nanofibers have the potential to serve as carrier matrix for bacteriocins and open a new field in developing controlled antimicrobial delivery systems for various applications.

## 1. Introduction

Bacteriocins (ribosomally synthesized antimicrobial peptides) and bacteriocin-like inhibitory substances (BLIS) are produced by several species of bacteria, including lactic acid bacteria that are generally recognized as safe [1–3]. Bacteriocins are diverse peptides and can be divided into the Class I (lanthionine-containing post-translationally modified bacteriocins), Class II (non-lanthionine-containing bacteriocins that do not undergo extensive post-translational modification) and the bacteriolysins [1]. Each of these groups can be further sub-divided. Bacteriocins are membrane active peptides and most form pores in the cell membranes of sensitive bacteria due to the cationic and amphiphilic nature of these peptides, however there are exceptions [4]. Pore formation leads to the disruption of the proton motive force (PMF) and the efflux of intracellular material [5]. Bacteriocins can have a narrow (active against closely related species) or broad spectrum of activity [6,7]. Class Ia bacteriocins, or lantibiotics, use lipid II as target for their mode of action and kill sensitive bacteria by pore formation or by hampering cell wall formation [8]. Lantibiotics can also inhibit the germination of spores [8] Class IIa, or pediocin-like bacteriocins, mainly form pores in the membranes of sensitive cells [1]. By disrupting the PMF, cells cannot secrete antibiotics via transport systems located in their cell membranes. This could be an important answer to our search towards more effective ways of infection control. More than 95% of *Staphylococcus aureus* isolates are resistant to penicillin. Of these, 60 to 70% are resistant to methicillin [9].

A few bacteriocins are active against methicillin-resistant strains of *Staphylococcus aureus* (MRSA), and *versus* other bacteria of interest in human health, such as *Pseudomonas aeruginosa*, *Streptococcus pneumonia* and vancomycin-resistant enterococci [10,11]. Bacteriocin ST4SA, produced by *Enterococcus mundtii*, is a Class IIa bacteriocin and is active against several pathogens, including *Acinetobacter baumannii*, *Pseudomonas aeruginosa*, *Streptococcus pneumonia*, *Staphylococcus aureus* and *Enterococcus faecium* [5]. Bacteriocin ST4SA survived conditions in the human and animal gastro-intestinal tract [12,13] and was constitutively expressed [14]. Nisin F, produced by *Lactococcus lactis*, was effective in the treatment of *Staphylococcus aureus* in respiratory tract infections [15]. Plantaricin 423, produced by *Lactobacillus plantarum*, displaced *Clostridium sporogenes* and *Enterococcus faecalis* from Caco-2 cells [16]. Plantaricin 423 is also a Class IIa bacteriocin and shows activity against *Bacillus cereus*, *Clostridium sporogenes*, *Enterococcus faecalis*, *Lactobacillus brevis* (*Lactobacillus* spp.), *Listeria innocua, Listeria monocytogenes*, *Propionibacterium* sp., *Propionibacterium acidipropionic* and *Staphylococcus carnosum* [17]. Bacteriocins may thus offer an alternative to control bacterial infections, administered alone or in combination with antibiotics [5]. The challenge would be to protect bacteriocins from proteolytic enzymes, keep the peptides in contact with the infected surface and extend the contact time for as long as possible.

Electrospun nanofibers, prepared from selected polymers, may be used as carrier matrix for bacteriocins and other antimicrobial peptides [18]. Nanofibers have been used to release antibiotics, proteins, growth factors, silver nanoparticles, plasmid DNA, as well as viable cells [18–26]. Specific structures can be designed, such as oxygen permeable membranes, matrices with a high surface to volume ratio and scaffolds that are morphologically similar to the extracellular matrix of skin [27–31].

In this paper we studied the feasibility of electrospinning bacteriocins into nanofibers, as well as investigating the nanofibers as a delivery system. Plantaricin 423 and bacteriocin ST4SA were used as model bacteriocins (Class IIa) in this study. These bacteriocins were dissolved in *N*,*N*-dimethylformamide (DMF) and electrospun into biodegradable nanofiber blends of poly(d,l-lactide) (PDLLA) and poly(ethylene oxide) (PEO). The nanofiber scaffolds were evaluated (*in vitro*) for the release of the peptides and antimicrobial activity.

## 2. Results

### 2.1. Electrospinning of Nanofibers

**Figure 1.**
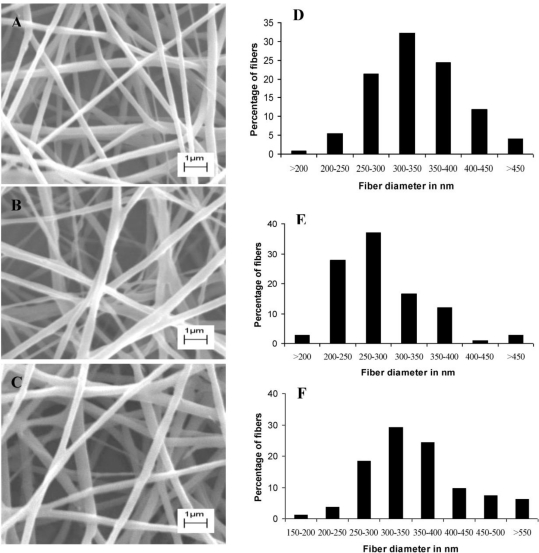
SEM images of electrospun nanofibers containing plantaricin 423 (**A**–**C**) and fiber diameter distributions of the electrospun nanofibers (**D**–**F**). (**A** and **D**) PEO 50:PDLLA 50, (**B** and **E**) PEO 70:PDLLA 30, (**C** and **F**) PEO 90:PDLLA 10.

Nanofibers produced from blends of PDLLA and PEO, containing plantaricin 423, have a regular texture and smooth surface, as revealed by scanning electron microscopy (SEM, Figure 1). The PEO 90:PDLLA 10 blend produced fibers ranging from 200 to 450 nm, with most of the fibers in the 300**–**350 nm range (Figure 1F). Fibers with similar dimensions were produced from the PEO 50:PDLLA 50 blend (Figure 1D). Fibers with slightly less variation (200–400 nm) and mostly in a smaller size range (250–300 nm) were produced from the PEO 70:PDLLA 30 blend (Figure 1E).

### 2.2. Fluorescent Microscopy Images of Polymers

**Figure 2.**
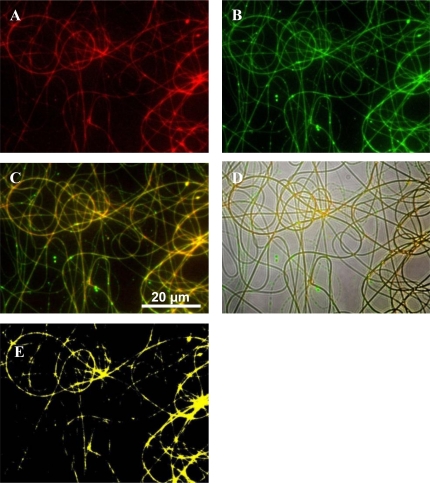
Fluorescent images of PEO and PDLLA labeled with RBITC and FITC, respectively. (**A**) Fluorescent image of PEO; (**B**) fluorescent image of PDLLA; (**C**) overlay of the fluorescent signals; (**D**) optical image of fibers with fluorescent signals; (**E**) co-localization of the two signals, indicating miscibility between PEO and PDLLA.

Fluorescent microscopy images have shown that PEO and PDLLA are homogenously distributed in the fiber structure and indicate a large degree of miscibility when used in a 50:50 combination (Figure 2). This blend was selected based on characteristics best suited for a drug delivery system, as discussed in this manuscript.

### 2.3. DSC Analysis

**Figure 3.**
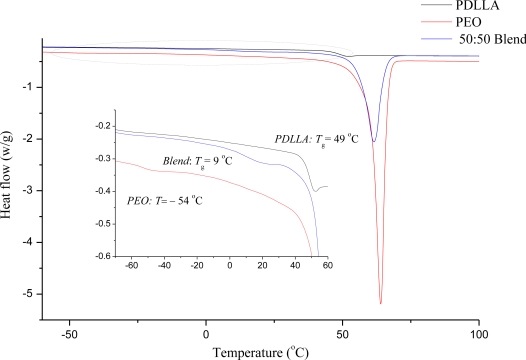
Differential scanning calorimetric (DSC) thermogram of the PEO 50:PDLLA 50 blend, as well as the component polymers, PEO and PDLLA.

It can be seen that the DSC curve of the PEO 50:PDLLA 50 blend yielded a single glass transition temperature (*T*_g_) of 9 °C, which falls between the *T*_g_ values recorded for PEO (−54 °C) and PDLLA (49 °C) (Figure 3). DSC curves provide a good indication of the macromolecular nature of the polymer, *i.e.*, crystallinity, *T*_g_ and crystalline melting point. In our study, PDLLA had no melt endothermic response as expected from an amorphous polymer, contrary to PEO where a melting endotherm was recorded at 64 °C. From the insert in Figure 3, it is evident that a single *T*_g_ was detectible at 9 °C, which falls between both *T*_g_s of PEO (−54 °C) and PDLLA (49 °C). It is therefore suggested that the blend is miscible in the amorphous phase. DSC analysis was also performed on the PEO 50:PDLLA 50 blend, containing plantaricin 423, and the *T*_g_ value decreased from 9 °C (in the blend without peptide) to −12 °C (data not shown). This *T*_g_ was still between that of the component polymers and therefore also suggested to be miscible in the amorphous phase.

### 2.4. Fluorescent Images of Labeled Polymers and Peptide

**Figure 4.**
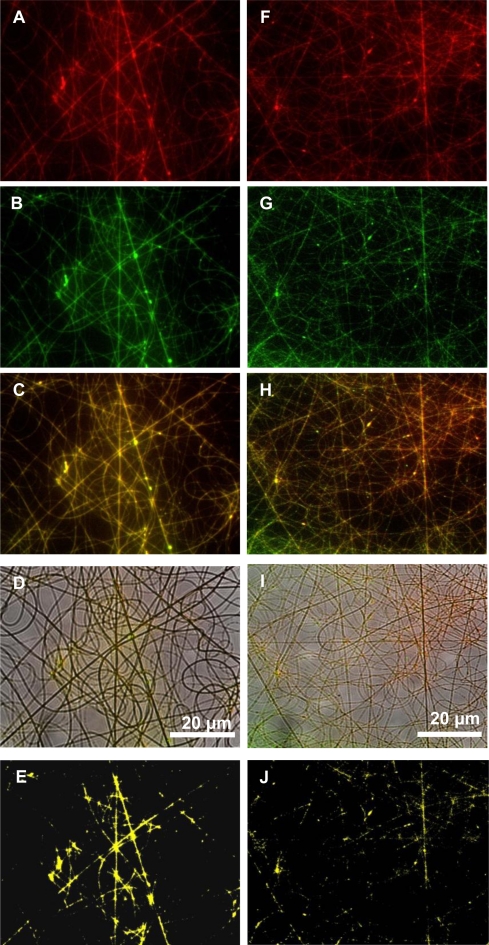
Fluorescent images of labeled plantaricin 423 and polymers electrospun into nanofibers consisting of PEO 50:PDLLA 50. (**A**) Fluorescent image of PEO labeled with RBITC in the fibers; (**B**) fluorescent image of the bacteriocin labeled with FITC in the fibers; (**C**) overlay of the fluorescent images; (**D**) optical image of fibers with fluorescent signals; (**E**) co-localization of the signals; (**F**) fluorescent image of bacteriocin labeled with RBITC in the fibers; (**G**) fluorescent image of PDLLA labeled with FITC in the fibers; (**H**) overlay of the two fluorescent images; (**I**) optical image of fibers with fluorescent signals; (**J**) co-localization of the two signals.

Fluorescently labeled plantaricin 423 was electrospun into nanofibers prepared from a PEO 50:PDLLA 50 blend. The bacteriocin was unevenly distributed in these fibers, indicating that the peptide has better interaction with one of the polymers (data not shown). Due to the amphiphilic nature of bacteriocins, we expected the peptide to show better association with one of the two polymers. Fluorescent images of nanofibers prepared from a PEO 50:PDLLA 50 blend are shown in Figure 4. Signals of the plantaricin 423 and PEO are overlapping (Figure 4C), suggesting that the peptide is associated with the polymer. This was verified with the co-localization of the signals (Figure 4E). These results were confirmed with fluorescent images of labeled plantaricin 423 and PDLLA. Green and red fluorescent signals could still be differentiated in the overlay, indicating that the peptide is closer associated with PEO. These results were supported with much less co-localization seen between the signals from the fluorescently labeled PDLLA and plantaricin 423 (Figure 4J).

### 2.5. Release of Antimicrobial Peptide

**Figure 5.**
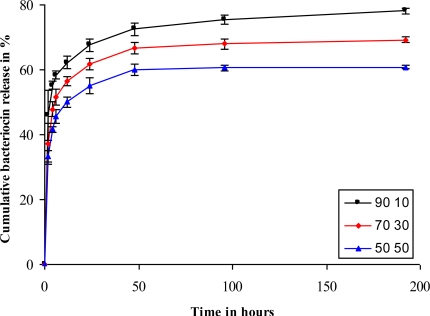
Cumulative release of plantaricin 423 from electrospun PEO:PDLLA blends. These fibers showed a high initial burst release and a more sustained release of bacteriocin over an 8 days period.

From cumulative release studies, it is evident that the PEO 90:PDLLA 10 blend released the highest concentration (78%) of the antimicrobial peptides (Figure 5). An initial release of 46% was recorded within the first 2 h, followed by 32% over the following 8 days (Figure 5). This could be ideal in the control of infections. A rapid release of antimicrobial peptides during the first 2 h would kill most of the pathogens, whereas a slow and constant release over the following few days would keep infection under control. The PEO 70:PDLLA 30 and PEO 50:PDLLA 50 blends yielded less promising results with 69% and 60% of the peptides being released over 8 days (Figure 5).

### 2.6. Morphological Changes and Weight Loss

**Figure 6.**
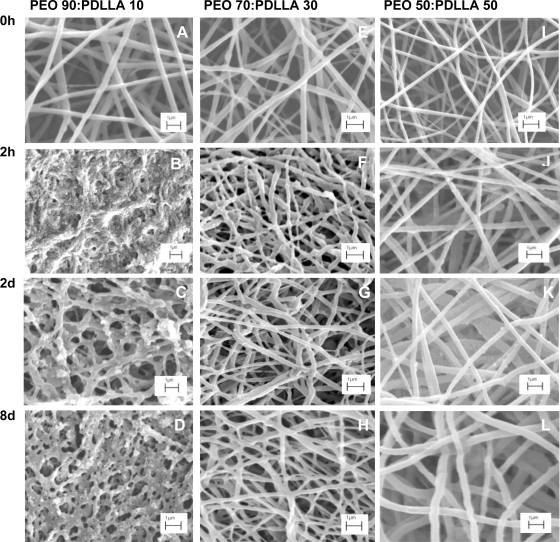
SEM images of PEO:PDLLA blend fibers, containing plantaricin 423, incubated in PBS buffer pH 7.4 for different time intervals. Significant morphological changes are seen in the PEO 90:PDLLA 10 blend (**A**–**D**). The PEO 70:PDLLA 30 blend showed some morphological changes; however the fiber structure stayed more or less intact (**E**–**H**). The PEO 50:PDLLA 50 blend showed very little morphological changes and the fiber structure stayed intact (**I**–**L**).

SEM images taken from PEO 50:PDLLA 50 nanofibers, containing plantaricin 423, after 8 days of incubation in PBS buffer (pH 7.4) did not reveal any significant structural changes or signs of deterioration of the fibrous structure (Figure 6I–L), suggesting that these nanofiber scaffolds would be stable for at least 8 days. Slight textural changes have been recorded for the PEO 70:PDLLA 30 blend, containing plantaricin 423, after 8 days (Figure 6E–H). However, significant morphological changes and signs of deterioration were observed for nanofibers prepared from the PEO 90:PDLLA 10 blend, containing plantaricin 423 (Figure 6A–D). Fibrous structures were visible, but most of the fibers formed a film-like structure after 2 h.

Nanofibers prepared from the PEO 90:PDLLA 10 blend showed high weight loss within the first few hours with only 24% of the original weight remaining. Eighty percent weight loss was seen over 8 days at 37 °C, whereas nanofibers prepared from the PEO 70:PDLLA 30 and PEO 50:PDLLA 50 blends lost approximately 60% and 40% of their original weight, respectively (data not shown).

### 2.7. Antimicrobial Activity Tests

**Figure 7.**
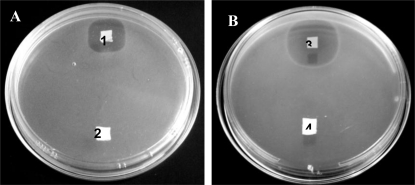
Antimicrobial activity tests of PEO 50:PDLLA 50 blend fibers containing plantaricin 423 (**A**) and bacteriocin ST4SA (**B**). Modified Kirby Bauer tests were performed by placing approximately 1 cm^2^ of fiber on plates seeded with target strains. *Enterococcus faecium* HKLHS and *Listeria monocytogenes* EGD-e served as target strains for plantaricin 423 and for bacteriocin ST4SA, respectively. A clear zone of inhibition can be seen surrounding the fibers containing bacteriocins after incubation. 1: PEO 50:PDLLA 50 blend fibers containing plantaricin 423, 2: PEO 50:PDLLA 50 blend fibers without plantaricin 423, 3: PEO 50:PDLLA 50 blend fibers containing bacteriocin ST4SA, 4: PEO 50:PDLLA 50 blend fibers without bacteriocin ST4SA.

**Figure 8.**
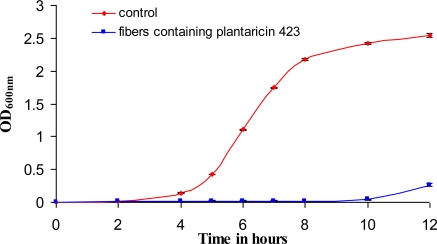
Growth inhibition of *Enterococcus faecium* HKLHS by PEO 50:PDLLA 50 blend nanofibers releasing plantaricin 423.

A clear zone of growth inhibition was recorded surrounding PEO 50:PDLLA 50 nanofibers for at least 6 days (Figure 7). This suggested that the nanofibers retained their antimicrobial activity for at least 6 days on solid medium. The peptides retained 88% of their original antimicrobial activity after 18 h of incubation at 37 °C in PBS pH 7.4. As little as 25 mg nanofibers inhibited the growth of *E. faecium* HKLHS in 50 mL MRS broth for up to 10 h, whilst cells in the control (without nanofibers) reached stationary phase after 10 h (Figure 8).

## 3. Experimental Section

### 3.1. Isolation of Antimicrobial Peptides

*Lactobacillus plantarum* 423 and *Enterococcus mundtii* ST4SA were cultured, separately, in MRS broth (Biolab, Biolab Diagnostics, Midrand, South Africa) for 24 h at 37 °C. Cells were harvested (8000× g, 10 min, 4 °C), the pH of the cell-free supernatant adjusted to between pH 6.5–7.0 with 10 M NaOH and then heated at 80 °C for 10 min to inactivate proteolytic enzymes. Peptides were precipitated from the cell-free supernatant with 70% saturated ammonium sulfate [32] and desalted against distilled water by using a 1000 Da cut-off dialysis membrane (Spectrum Inc., CA, USA). Protein concentrations were determined by using the Micro-bicinchoninic Acid (BCA) kit (Pierce, Rockford, IL). Readings were recorded at 562 nm. Samples were freeze-dried and stored at −20 °C.

### 3.2. Electrospinning of Nanofibers

Freeze-dried peptides were dissolved in *N*,*N*-dimethylformamide (DMF) and centrifuged (6000× g, 1 min) to obtain the supernatants. From each of these supernatants, 2 mL was used as solvent for PDLLA (20–24%, w/v, Sigma-Aldrich) blended with poly(ethylene oxide) (PEO, 200,000 Da, Sigma-Aldrich) at a ratio of 10:90, 30:70 and 50:50, respectively. Solutions were heated to 40 °C on a hot plate and then electrospun using a gravity system described by [20]. A constant electric field of +10 kV was applied to the polymer solution and −5 kV to the collector. The collector was placed 15 cm from the polymer solution. The relative humidity was kept constant between 50–55%.

### 3.3. Scanning Electron Microscopy of Electrospun Fibers

Images of the nanofibers were recorded with a Leo^®^ 1430VP Scanning Electron Microscope (SEM). Samples were coated with a thin layer of gold to increase conductivity. Fiber diameter was determined by using the SEM Image Studio Software (version 10.1).

### 3.4. Fluorescent Labeling of Antimicrobial Peptides and Polymers

Freeze-dried plantaricin 423 (20 mg) was suspended in 1 mL DMF and the supernatant was reacted with 20 mg rhodamine B isothiocyanate (RBITC) or fluorescein isothiocyanate (FITC) from Sigma-Aldrich, dissolved in the same volume. The solution was placed on an orbital shaker and incubated in the dark at 4 °C for 48 h. Unbound fluorescent marker was removed by dialyzing against sterile distilled water for 3 weeks at 4 °C. Dialyses bags with 1000 Da cut-off (Spectrum Inc., CA, USA) were used. Labeled peptides were freeze-dried, dissolved in DMF and electrospun into nanofibers as described before.

PEO (0.2048 mg) was dissolved in 2 mL sterile distilled water containing RBITC (20 mg/mL). The polymer solution was placed on an orbital shaker and incubated in the dark at 25 °C for 48 h. Unbound fluorescent marker was removed through dialysis as described before and freeze-dried. PDLLA (0.2048 mg) was dissolved in 2 mL DMF and was labeled with FITC (20 mg/mL) for 48h on an orbital shaker and incubated in the dark at 25 °C.

The labeled peptides and polymer solution were electrospun into nanofibers as described before. Nanofibers were excited at 472 nm and 572 nm, respectively, by using a Xenon-Arc burner light source (Olympus Biosystems GMBH). Fluorescent images were captured with an Olympus Cell^^R^ system attached to an IX-81 inverted fluorescent microscope. An Olympus Plan Apo N 60×/1.4 Oil objective was used. Images were taken with an F-view-II cooled CCD camera (Soft Imaging Systems) and the background subtracted using the Cell^^R^ imaging software.

### 3.5. Differential Scanning Calorimetry (DSC)

Thermal analysis was carried out with a TA Q100 Differential Scanning Calorimeter (DSC) (TA Instruments) which was calibrated against an indium standard. An empty aluminium pan was the reference. Fiber samples (15–18 mg) were run at a 10 °C/min in three cycles under nitrogen atmosphere (flow rate 50 mL/min). In the first cycle, samples were heated from −80 °C to 180 °C and isothermed for 1 min; cooled back to −80 °C and isothermed for 5 min. Finally, the samples were heated for a second time at 10 °C/min to 180 °C. The crystalline melting temperature (*T*_m_) and the glass transition temperature (*T*_g_) were recorded in the second heating cycle.

### 3.6. Release of Antimicrobial Peptides and Degradation of Nanofibers

Nanofibers (25 mg) were placed in 2 mL sterile phosphate buffered saline (PBS, pH 7.4) and incubated at 37 °C on an orbital shaker (30 rpm). At pre-determined time intervals the PBS was replaced with 2 mL sterile PBS (pH 7.4). The protein concentration in each sample was determined by using the BCA protein assay kit as described before. The concentration of bacteriocin in the nanofibers was determined by dissolving 25 mg fibers in DMF. This suspension was diluted in PBS 7.4 before the protein concentration was determined by using the BCA protein assay kit as described before. Nanofibers were freeze-dried and then visualized by SEM. Changes in the weight of nanofibers were recorded.

### 3.7. Antimicrobial Activity Tests

In a separate experiment, 25 mg of PEO 50:PDLLA 50 nanofibers containing plantaricin 423 was placed in 50 mL MRS broth, inoculated with *Enterococcus faecium* HKLHS (10^5^ cfu/mL) and incubated at 37 °C for 12 h. Changes in optical density readings were recorded at 600 nm.

## 4. Discussion

Electrospinning is a versatile technique to produce large amounts of fibers, in the micrometer to nanometer range, in a short amount of time. A variety of natural and synthetic polymers have been electrospun into fibers and some have been used to control the release of various compounds [13,19,22,26,31,33]. Blending hydrophilic and hydrophobic polymers directly in a suitable solvent before electrospinning, is an easy way to change the hydrophobicity, thermal stability and mechanical strength of the fibers [34]. Since blending is an attractive way to modify polymer properties, this method was chosen to modify the hydrophobicity/hydrophilicity of the electrospun fibers by the addition of PEO, to help facilitate the release of the bacteriocin from the nanofibers.

Fibers were all in the nanometer range and addition of bacteriocin did not influence the fiber structure as can be seen in Figure 1.

Polymers were labeled to elucidate fiber morphologies after electrospinning. Fluorescent microscopy images showed that there is a large degree of miscibility between the PEO and PDLLA when these polymers are used in a 50:50 combination (Figure 2). However, to confirm these results and to determine the miscibility of the polymers, DSC analysis was performed on the PEO 50:PDLLA 50 fiber blend, see Figure 3. This fiber blend was chosen as it retained its structure the best, had the least amount of weight loss and still released enough bacteriocin to inhibit the growth of a sensitive strain. Nijenhuis *et al.* [34] have studied thermal properties using DSC of polymer blends containing various ratios of PEO and the poly l-lactide (PLLA) isomer. The results showed that blends containing up to 50 wt% PEO were miscible in the amorphous phase. In this work, PDLLA which is an amorphous grade unlike the semi crystalline poly (d-lactide) PDLA was used. A good indication of a miscible polymer blend is the shift of the *T*_g_ to a value between the *T*_g_s of the pure polymers. The *T*_g_ value of a mixed phase is determined by the weight fraction of each polymer in that phase, and by the *T*_g_s of the pure polymers. DSC curves of the second heating cycle of the PEO 50:PDLLA 50 blend and the corresponding homopolymers are shown in Figure 3. A single *T*_g_ (9 °C) can be seen that is between the *T*_g_s values for PEO (−54 °C) and PDLLA (49 °C) which suggests that the PEO 50:PDLLA 50 blend is miscible in the amorphous phase. DSC analysis performed on the PEO 50:PDLLA 50 blend, containing plantaricin 423, revealed that the *T*_g_ value still fell between the *T*_g_ of the component polymers and is therefore still miscible to an extent. It could be due to the peptide that is more associated with the PEO domains or simply restricting chain mobility. Investigation of the distribution of bacteriocins in the final fiber structure after electrospinning could yield important information in designing polymeric drug delivery systems for these peptides.

Fluorescent microscopy images revealed that the bacteriocin was better associated with PEO than with PDLLA, even though the peptide was associated with both these polymers (Figure 4). Bacteriocins are amphiphilic peptides with both hydrophilic and hydrophobic residues [1]. Because of this amphiphilic nature it is possible for the peptide to interact better with one polymer than with the other. These results indicate that the bacteriocin is better associated with the hydrophilic polymer, in this case PEO, but were also associated with the PDLLA to a lesser extent. This would explain the high initial burst release within the first few hours.

Cumulative release studies revealed that fibers with a higher PEO content showed a higher release of the bacteriocin than those with a lower PEO content (Figure 5). An initial burst release was seen within 2 h, after which a more sustained release was seen. This could be an ideal release profile in the control of infections. A rapid release of antimicrobial peptides during the first 2 h would kill most of the pathogens, whereas a slow and constant release over the following few days would keep infection under control. The PEO 70:PDLLA 30 and PEO 50:PDLLA 50 blends released lower amounts of the peptide, with the PEO 70:PDLLA 30 and PEO 50:PDLLA 50 blends releasing 69% and 60% of the peptides, respectively (Figure 5). Blends of different polymers have earlier shown to facilitate the release of molecules from electrospun fibers. Lysozyme release from nanofibers was facilitated by blending PEO and poly(ɛ-caprolactone) (PCL), with a 90:10 blend releasing most of the lysozyme with a high initial burst release [22]. Higher concentrations of cytochrome C were also released from fibers consisting of poly(l-lactide) PLLA and high concentrations of both poly(l-lysine) (PLL) and poly(ethylene imine) (PEI), respectively [26]. Fibers containing 50% PLL released most of the protein (75%) with a high initial burst release.

SEM images revealed that as the PEO content increased, significant morphological changes can be seen as the incubation time increased (Figure 6) The PEO 90:PDLLA 10 blend showed the most significant morphological changes over the 8 day period. This could be due to the high content of PEO in the fibers as PEO swells readily in an aqueous environment and dissolves easily. The resulting destruction of the nanofiber scaffold explains the higher release of antimicrobial peptide as observed over 8 days (Figure 5). Fibers retained their structure better in the PBS buffer (pH 7.4), as the PEO content in the fibers decreased. This can be observed in the PEO 50:PDLLA 50 blend, with this blend showing no significant signs of structural changes or deterioration in the buffer medium (Figure 6I–L). Slight structural changes were seen for the PEO 70:PDLLA 30 blend after 8 days (Figure 6E–H). These results suggest that the PEO 50:PDLLA 50 blend nanofibers would be stable for at least 8 days and show no significant signs of degradation. The ideal nanofiber drug delivery systems has to retain its structure, have high oxygen permeability, variable pore size and a high surface to volume ratio. Nanofibers prepared from the PEO 90:PDLLA 10 blend showed very high weight loss over the 8 days period, which is not very desirable. The PEO 70:PDLLA 30 showed lower weight loss, however the PEO 50:PDLLA 50 showed the best results and lost the least amount of its original weight. It is very important that the nanofiber scaffolds retain their integrity and with this in mind, the PEO 50:PDLLA 50 would be the preferred choice as drug delivery system in this work.

Bacteriocins retained their activity after electrospinning and were able to inhibit the growth of a sensitive strain on solid media as well as in liquid broth (Figures 7 and 8). The bacteriocins showed activity on the solid media for at least 6 days of incubation. Growth inhibition in liquid broth was observed and may be obtained over longer periods of time by electrospinning higher concentrations of bacteriocin in the fibers, by using more fibers (weight), or by replacing the fibers in the broth with new fibers at certain time intervals. Only 25 mg of fibers, containing plantaricin 423, was able to inhibit actively growing cells of *Enterococcus faecium* HKLHS, indicating the potent antimicrobial effect of this bacteriocin. The difference in antimicrobial activity and release on solid media and in liquid broth gives an indication that there is a big difference in release profile on solid media and in a buffer/liquid medium. During release studies in a buffer medium, a high concentration gradient is created (such as in the case of the method used for determining release) by replacing the old buffer with new buffer at each sampling point. This can increase the rate of diffusion of the peptide from the nanofiber scaffold to the buffer medium. The detection limit of the protein assay is also in μg/mL, whereas the bacteriocins can still be active at lower concentrations of ng/mL. However, the concentration gradient can be much lower on solid media, resulting in lower release profiles and thus leads to increased periods of antimicrobial activity.

## 5. Conclusion

Bacteriocins remained active after electrospinning into nanofiber scaffolds. This technique provides the option to electrospin high concentrations of antimicrobial peptides into nanofibers. The rate at which these peptides are released is controlled by selection of the correct combination of polymers. The degradation rate and mechanical properties of PDLLA can be improved by blending with a hydrophilic polymer such as PEO. Antimicrobial peptides electrospun into nanofibers may have potential applications in the pharmaceutical and food industries to control microbial growth.
